# A multi-modal data fusion and real-time monitoring on stroke risk prediction using federated learning

**DOI:** 10.1371/journal.pone.0330244

**Published:** 2026-04-06

**Authors:** Ramya Sree K, Mohan Kumar P

**Affiliations:** School of Computer Science and Engineering, Vellore Institute of Technology, Vellore, Tamil Nadu, India; Incheon National University, KOREA, REPUBLIC OF

## Abstract

Predicting the risk of stroke is one of the critical problems in healthcare, which necessitates efficient solutions for providing accurate and prompt risk assessments while preserving data confidentiality. This work proposes a new framework using Federated Learning (FL) to combine Multi-Layer Perceptron (MLP) and Gated Recurrent Unit (GRU) models that are essential in analyzing multimodal data. Implemented in Python, the approach incorporates two datasets: Dataset 1, which consists of Demographic data medical history, and lifestyle data, and the second dataset, which includes the normal condition and the affected stroke condition CT scan images. Imputation of missing values, feature normalization by Min-Max scaling, and handling of imbalanced classes with SMOTE make the data pre-processing procedures exhaustive. In FL architecture three clients –Client A, Client B, and Client C – process a split multimodal dataset containing static and sequential information. Each client independently trains an MLP-GRU model. Each is applied with MLP handling static features from Dataset 1 and GRU handling sequential features from Dataset 2. To update models, Federated Averaging is used on a central server, to create a global model that is then returned to the clients for further refinement. The accuracy of the proposed method averages 99.00% and surpasses other models by 2.5% including CNN, LSTM, Random Forest, and SVM. By enhancing MLP with GRU and applying them to a privacy-preserving FL framework,The study addresses the fragmented use of multimodal medical data, where clinical records and imaging are generally evaluated separately, resulting in inadequate diagnostic support. The strategy integrates complementary modalities to create a more comprehensive perspective of patient health, enhancing healthcare predictive accuracy and decision-making. This incentive is essential for improving computational methods and linking technical advancement with medical objectives like fast diagnosis and therapy planning. The introduction emphasises the therapeutic necessity of harmonising organized and unstructured data to reduce diagnostic ambiguity. A translational approach is used to discuss how multimodal integration might improve clinical workflows, develop collaborative healthcare systems, and support sustainable medical practices. This repeated emphasis links methodological advances to real-world healthcare issues, boosting the study’s academic relevance sets.

## 1. Introduction

A stroke is a condition that arises when the flow of blood to a part of the brain is disrupted or reduced, depriving the tissue of the brain of the oxygen and nutrients it needs [1]. Because stroke victims’ brain cells begin disintegrating within minutes, it is an acute condition that has to be treated quickly to reduce brain damage and any repercussions. There are two main types of strokes: ischemic stroke, that is caused by blockages or constriction in the arteries leading to the brain, and hemorrhagic stroke, that is caused by blood vessels burst and spill blood through or towards the brain [2]. A mini-stroke, also referred to as a transient ischemic attack, is a third ailment that is characterized by a brief period of stroke-like symptoms. The most frequent kind of stroke, ischemic stroke, is brought on by fatty deposits or blood clots that block the blood arteries in the brain. In contrast, hemorrhagic strokes occur when blood vessels within the brain burst, frequently as a result of arteriovenous malformations, aneurysms, or excessive blood pressure. The less severe TIAs (Transient ischemic attack) are caused by temporary clots and serve as a warning sign for potential future strokes [3]. Diabetes, elevated blood pressure, obesity, smoking, high cholesterol, as well as a family association with stroke are risk factors for strokes [4].

Numbness or weakness, particularly on a single side of the body, disorientation, trouble speaking and comprehending speech, vision issues, dizziness, trouble with balance, and a severe headache are some of the signs of a stroke that frequently manifest abruptly. It is critical to identify these symptoms as soon as possible because the results can be greatly impacted by prompt medical intervention [5]. Physical examinations as well as imaging tests, including CT, MRI, and ultrasound scans, are commonly used in diagnosis. These assessments aid in identifying the kind of stroke as well as the best course of action. The treatment of stroke depends on its type [6]. Hemorrhagic strokes require different approaches, such as controlling bleeding and reducing pressure in the brain through surgical interventions. Occupational, speech, and physical therapy are examples of rehabilitation therapies used in post-stroke care with the goal of restoring lost functions and enhancing quality of life. Medications to manage risk factors, like antihypertensives and anticoagulants, are often prescribed to prevent recurrent strokes.

Important measures include keeping a healthy weight, avoiding smoking as well as excessive alcohol consumption, engaging in regular physical activity, and eating a balanced diet high in vegetables and fruits and low in saturated fats. Stroke risk can also be decreased by managing long-term illnesses like high blood pressure, diabetes, and cholesterol through medication and routine checkups [7]. In order to promote healthy lifestyle choices and increase awareness of stroke prevention, public health efforts and education campaigns are essential. Advancements in medical research and technology are continuously improving stroke care and outcomes. Emerging treatments, such as neuroprotective agents and advanced clot retrieval devices, are under investigation. Furthermore, the use of AI and machine learning for healthcare is improving the capacity to forecast the danger of stroke, customize treatment regimens, and keep an eye on patients in real time [8]. Specialized stroke care is becoming more widely available because to telemedicine, especially in rural and underdeveloped areas. Continued investment in research, public health strategies, to further lessen the toll that stroke takes on both people and society as a whole, healthcare infrastructure is crucial.

Prior research on stroke risk prediction has revealed several important drawbacks, such as the underperformance of pooled cohort equations and traditional stroke-specific algorithms in racially diverse populations, especially Black people, and the dependence on additive and linear models that are unable to adequately capture the intricate interactions between a variety of risk factors. In addition, these models are not easily explainable, which can be disadvantageous for use in clinical programs, and their performance in general is often prejudiced by the over-emphasis of measurements carried out on other datasets. Although the extent of use of machine learning methods has led to improvement on the predicting accuracies, they are still seen as having problems in handling datasets and adequately addressing multimodal conditions. As for these drawbacks, we propose Integrative Artificial Intelligence for Multi-Modal Data Integration, Real-Time Monitoring through Federated Learning, and Stroke Risk Prediction. Due to the lack of precision in other models of facilitating stroke risk assessment, this study aims to develop a masculine AI model that will use data from different sources, including patient records, lifestyle factors and real-time data. We ensure constant risk evaluation as well as patients’ confidentiality by place into operation, which enables quick actions, and federated learning. Our approach is expected to discover new complex relationships and model-specific risk patterns that would improve preventive measures for stroke and address the differences documented in previous work [9].

By identifying those who are at increased risk before they have a stroke, stroke risk predicting plays a critical role in minimizing this crippling disorder. Timely measures made possible by early identification can greatly lower the risk of a stroke [10]. This proactive approach is essential as strokes often result in severe physical and cognitive impairments, and in some cases, can be fatal. By accurately predicting stroke risk, healthcare providers can tailor prevention strategies to individual patients, thereby improving outcomes and reducing the overall burden on healthcare systems [11]. Traditionally, stroke risk prediction has relied on clinical assessments and risk factor scoring systems. Risk is assessed using tools like the Framingham Stroke Risk Profile as well as the CHA2DS2-VASc score, which take into account variables including age, smoking, diabetes, hypertension, and a history of cardiovascular illnesses [12]. While these tools provide valuable insights, they often lack precision due to their reliance on broad, population-based data. These methods can miss nuanced interactions between various risk factors and may not fully capture an individual’s unique risk profile, leading to either overestimation or underestimation of stroke risk.

With the advent of big data and advanced analytical techniques, there has been a significant shift towards more sophisticated stroke risk prediction models. These intricate datasets can be analyzed by machine learning algorithms to find connections and patterns that conventional approaches might miss [13]. This multi-modal method enables a more thorough evaluation of a person’s risk of stroke, considering a broader range of variables and their interactions. Artificial intelligence and machine learning have revolutionized stroke risk prediction by enabling the development of predictive models that can learn from vast amounts of data [14]. Methods like neural networks, deep learning, and ensemble methods are particularly effective in handling large and diverse datasets. By learning from fresh data, AI models may consistently increase the accuracy of their predictions, making them highly adaptable to evolving healthcare patterns. These models can provide personalized risk assessments, helping clinicians to develop targeted prevention and intervention strategies tailored to each patient’s specific risk profile [15].

In the case of using multi-modal data for the prediction of risks of stroke, one major issue is privacy and protection of the patient’s data [16]. This is solved in federated learning where models are trained on the decentralized data in as many healthcare facilities as possible without necessarily exposing patients’ details to other healthcare institutions. In this style, the local institutions build the local models and update only the model parameters in a central server, which fuses them to develop a world model [17]. This method avoids the use of identification information but uses other related data to improve the predictive ability. Federated learning helps to enhance the patient’s data privacy and protection since the data does not go through a central point that makes it exposed and tends to contravene the data protection laws. The future of stroke risk prediction lies in the continued integration of advanced technologies and personalized medicine. Combining genetic data, biomarkers, and real-time physiological monitoring with AI-driven predictive models holds promise for even more accurate and early detection of stroke risk. Additionally, the implementation of real-time monitoring systems can provide continuous risk assessment and timely alerts, enabling immediate interventions. To solve ethical, legal, and technical issues, cooperation between legislators, healthcare professionals, and technology developers will be crucial. These developments could revolutionize stroke prevention as they gain traction, improving patient outcomes and lowering medical expenses.

The key contributions of the paper is summarized in the following points

- The paper introduces a novel stroke risk prediction methodology that combines MLP for analysing static features and GRU for processing sequential imaging data. This integration leverages the strengths of both architectures to enhance predictive accuracy.
- By using a federated learning architecture, the methodology allows several healthcare organizations to work together to train the global framework without exchanging raw data. While still taking advantage of the various data sources found in different organizations, this method guarantees data confidentiality and privacy.
- The paper addresses data pre-processing rigorously, including imputation for missing values, Min-Max scaling for normalization, and SMOTE to handle class imbalances, particularly in the imaging dataset. These steps ensure high-quality input data for model training.
- The clients process the multimodal dataset by splitting it into three equal parts, containing both static and sequential features. Each client trains an MLP-GRU model and sends updates to a centralized server. These updates are then combined by the server using Federated Averaging to produce a global model, that is then dispersed for additional local training.
- The federated learning setup allows for iterative enhancement of the global model. Local model updates from different clients are aggregated using techniques like Federated Averaging, maintaining data privacy while consistently enhancing the model’s performance. Strong and accurate stroke risk estimations are guaranteed by this iterative procedure.

The following is how the paper is organized: Section 2 discusses the body of research on risk of stroke prediction techniques, with an emphasis on federated learning and integrative AI. Section 3 provides the Methodology introduces the proposed methodology, including data pre-processing, model architecture (MLP-GRU), and federated learning framework. Section 5 gives the Results and Discussion. Section 6 highlights the importance of the suggested method in stroke risk prediction, summarizes results, and makes recommendations for future research areas.

Multimodal fusion improves diagnosis accuracy by combining imaging and clinical variables, robustness against missing data, and privacy-preserving training through federated setups. The computational requirement and multimodal contribution balance difficulty are acknowledged. This balanced treatment helps readers understand the unique and practical aspects of the proposed techniques.

## 2. Related works

Ngamdu and Kalra [18] show that after a cerebrovascular insult, a patient can experience the manifestation of one or another form of CI, from mild CI to dementia. At plaque level, atherosclerosis has been correlated with likelihood of stroke in carotid arteries, CI, and dementia in coronary and breast arteries. This review is restricted to relationship between stroke, carotid intima-media thickness (CI), and dementia risk in addition to subclinical atherosclerotic calcification in these arteries as determined by computed tomography. The main strength of CT is the possibility of determining the extent of the calcification of the blood artery segments in different segments, only once. However, the degree of association between CT results and CI and stroke depends on the location and extent of arterial damage. However, there are not many studies in this field yet, therefore more thorough research is needed to address the issue of people with subclinical vascular disease having a higher risk of cognitive decline. Additionally, it is critical to evaluate the effectiveness of the preventive interventions among incidental VCs management in patients through RCT to compare the incidence of incidents such as stroke and CI.

Bârsan et al [19] explains Using demographics, clinical, laboratory, and imaging data a three-year risk score of death in patients with AIS was developed and externally validated within the context of the current study The study sample was made up of 244 AIS treated in a tertiary care setting and followed up over three years. Potential predictor variables included in the data were demographics, clinical status, leptin and resisting test results, and imaging characteristics. The participants were divided into two groups: Validation of 80 facilities and 164 for prediction purposes formed the respective groups. The following factors were independently linked to three-year mortality: surface lesions in carotid arteries, low haemoglobin, high resistance levels, increased age, and a higher score at NIHSS test. These were added to the prediction model and the model was then tested on another sample. In this investigation, leptin levels were not a biomarker predictive for mortality in either a secondary manner. We developed and externally validated a new and credible prognostic index for three-year mortality in AIS patients. Moreover, the present study identified several traits that have been proven individually associated with increased long-term mortality.

Vu et al [20] aimed during the exploration of the possibilities of applying concepts of machine learning for early detection of strokes and estimation of other vital risk factors with the help of data collected from the Suita trial which includes 53 factors and 7,389 volunteers. First, the participants were partitioned into risk clusters by applying k-prototype clustering technique. The prediction of stroke outcomes was then done using five supervised algorithms: Other methods include support vector machines, logistic regression, random forests, extreme gradient boosting, and light gradient boosting. There is sufficient statistical evidence in relation to the findings based on the unsupervised clustering approach to suggest that the detected groups of risk contribute to stroke different rates that are significantly distinct. RF’s objective was set to produce the highest predictive measures among all supervised learning methods. As for high-risk population subgroup, the above-mentioned indices of SHAP analysis indicated that blood glucose, metabolic syndrome, age, systolic blood pressure, hypertension, eGFR, and increased clustering according to the suggested UMAP method were independent risk factors for stroke. In addition, from the biomarkers of the present study, this study proposed new indices for prediction of a stroke event by calculating optimizing calcium level, haemoglobin, fructosamine and thickness of the elbow joint. As a result, machine learning allowed for making stroke risk prognoses with high accuracy and identifying new biomarkers; thus, the relations between the variables were quantitatively described in the context of stroke risk assessment and biomarker analysis.

Zhou et al [21] Traditional centralized computing techniques can consume large amounts of server storage, network bandwidth, and processing power on the central server in the present field of risk for diseases prediction research. Create federated learning, a distributed intelligence edge computing technique, to forecast disease risks in response. Then also propose an effective technique that enables training and inferring the prediction models directly at the edge without escalating the examination storage. Using this method minimizes the usage of network bandwidth and server computation time. To this end, we built a new simple neural network model known as the Linge (Lightweight Neural Network Models for the Edge). When operating in memory, this model requires 155.28 MB, although having just 7.63 MB of parameters. Experiments comparing the Linge model with the EfficientNetV2 model showed an improvement in performance. This experiment proves that the Linge model is capable of performing disease risk predictions at edge with low computational cost but higher accuracy.

Shen et al [22] In order to quantify the severity risk of FR and risk assessments both before and after EVT, the project aims to develop and test a novel scoring system called the Futile Recanalization Prediction Score (FRPS). Specifically, the FRPS was established through a formal process of screening the predictor variables according to clinical relevance and influence. Prior meta-analysis was used to create the initial equations, which were subsequently optimized using other statistical analyses. There were nonlinear relationships between the variables and for that machine learning, especially, random forest regression was employed. To check conventional validation and model fitness, a framework of 5 fold cross validation was used. Age at stroke, sex, AF, HTN, DM, hyperlipidemia, severe memory impairment prior to stroke, pre-stroke mRS, admission SBP, onset-to-puncture time, sICH, as well as NIHSS were among the factors included in the final 28-variable FRPS model. The mean R-squared that was obtained while reporting the random forest model’s results was roughly 0.992. FRPS scores were categorized as mild if they were less than 66, moderate if they were between 66 and 80, and severe if they were greater than 80. Thanks to the FRPS, clinicians can find useful information for the planning of the further therapy and the patient management describing the probability of the severe risk of futile recanalization. This tool may assist in identifying patients that will benefit most from EVT and increase the predictive ability of mortality after EVT has been performed. Additional clinical assessment in other health care facilities is needed to determine the applicability, efficiency and credibility of the created model.

Together, the reviewed papers demonstrate advances in stroke risk estimation using a variety of approaches, with a special emphasis on machine learning as well as genetic evaluation to overcome shortcomings in conventional models. In order to address racial inequities, one study contrasted cutting-edge machine learning techniques with classic stroke risk assessments. The results showed that the old methods functioned poorly in Black populations, underscoring the need for more diverse risk factors and advanced modeling. An additional investigation carried out a genome-wide association study spanning several ancestries, unveiling novel genetic loci associated with stroke and proposing prospective therapeutic targets. However, the study’s dependence on meta-analyses may restrict its relevance to non-European populations. Furthermore, through the incorporation of comprehensive concurrent illnesses, a study employing machine learning algorithms, such as logistic regression, demonstrated enhanced stroke prediction in patients with non-anticoagulated atrial flutter; however, the study’s reliance on particular health plan data may limit its generalizability. A perceptron neural network employing these factors surpassed other approaches, according to another study that used principal component analysis using electronic medical information to determine critical stroke risk factors. The model’s usefulness in practice, however, can be impacted by the use of subsampling techniques to balance the dataset. Random Forest was shown to be the most accurate, although possible overfitting and interpretability difficulties were noted. Finally, new risk profiles and associations were found in research that sought to improve the Framingham Stroke Risk Scores using a non-linear model and Optimal Classification Trees. However, the intricacy of non-linear interactions may make practical deployment difficult. Taken together, these investigations demonstrate the potential for enhanced stroke prediction using sophisticated machine learning and genetic techniques, but also draw attention to issues with data variety, generalizability, and model interpretability.

The discussion includes two key literature contributions that support the methodology. The first, FT_ANPD: A dual-feature two-sided attention network for anticancer natural products detection, shows how attention mechanisms can represent complicated biomedical interactions and the importance of feature-level interpretability sets. The second, Incorporating Part-whole Hierarchies into Fully Convolutional Networks for Scene Parsing, shows how hierarchical modeling improves image-based contextual understanding. The study links the suggested method to deep learning research on attention, hierarchy, and multimodal integration sets by referencing existing works.

## 3. Integrative artificial intelligence for stroke risk prediction

MLP and GRU are employed in the machine learning framework for the suggested stroke risk prediction approach, which would enable effective utilization of various data sets while also guaranteeing patient data confidentiality and privacy. Initially, two distinct datasets are used: Demographic, medical, and lifestyle data described in Dataset 1 and CT scan images as a part of Dataset 2, which shows the normal and stroke-affected conditions. These datasets are preprocessed individually to modify missing values and scale the numerical features in the datasets using imputation and Min-Max scaling respectively. For dealing with the class imbalance issue particularly Dataset 2, the SMOTE technique is applied. In the case of federated learning, there are three clients, namely Client A, Client B, and Client C and each client uses their own dataset to train the local model. The clients distribute the multimodal dataset into three equal parts and process them. The multifaceted data set contains quantitative and temporal aspects. Clients come up with their local model (MLP-GRU) and then send updates of that model to a central server, which uses methods such as Federated Averaging to build a global model without moving data. This aggregated model is then broadcast to the other local clients in order to improve the circulated model periodically. Considering that, MLP is suitable for static data analysis while GRU can handle the sequential data, by incorporating the federated learning approach, the proposed approach provides a complete, reliable, and non-disclosure manner for stroke risk prediction. It allows healthcare institutions’ collaboration and early identification of high-risk patients with individualized treatment plans, solving the technical and ethical issues of modern medical data analysis for stroke.

**Fig 1:**
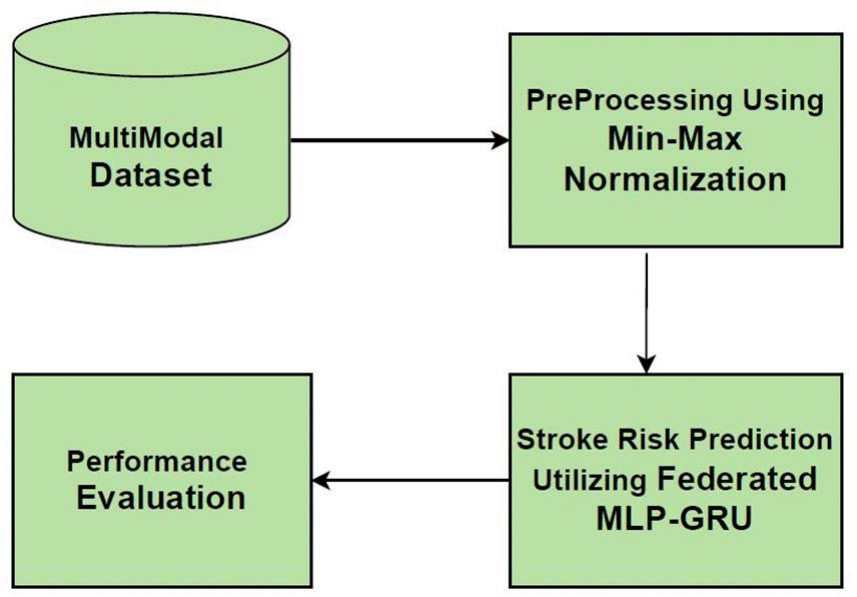
Overall Architecture of the Proposed Method.

Fig 1 shows overall Architecture of MultiModel Dataset,PreProcessing Techniques with MLP-GRU Using Federated Learning along with Performance Evaluation

### 3.1 Data collection

#### 3.1.1 Dataset 1.

The provided dataset, which was acquired from Kaggle, attempts to determine the patient’s risk of having a stroke by using the parameters that ‘https://www.kaggle.com/datasets/fedesoriano/stroke-prediction-dataset’. The WHO reports that stroke is at least responsible for 5.5 million deaths globally, putting it second as a leading cause of death in the world. A unique identification, gender, age, hypertension, heart disease type, marital status, occupation, and place of residence are additional characteristics contained at the patient level. Additionally, in addition to lifestyle factors like smoking status, it offers clinical indicators such as average blood glucose levels and body mass index, or BMI. However, if the patient has had a stroke, it is the mentioned goal variable. It is crucial to have extensive data that defines the patient’s lifestyle, as well as his/her medical and demographic history to generate prediction models to foresee the possibility of a stroke condition at a preliminary stage. Table 1 below gives a description of the stroke dataset like Attributes,values/Types.

**Table 1:** Stroke dataset and their values/types.

Attribute	Description	Values/Type
Id	Unique identifier	Integer
Gender	The patient’s gender	“Male”, “Female”, “Other”
Age	The patient’s age	Float
Hypertension	Hypertension status	0 (no), 1 (yes)
heart disease	Status of heart disease	0 (no), 1 (yes)
ever married	Status of marriage	“No”, “Yes”
work type	Type of employment	“children”, “Govt_*j*_*ob*”,”*Neverworked*”,”*Private*”,”*Sel f* −*employed*”
Residence type	Type of residence	“Rural”, “Urban”
avg glucose level	Average blood glucose level	Float
Bmi	Body Mass Index	Float
smoking status	Status of smoking	“formerly smoked”, “never smoked”, “smokes”, “Unknown”
Stroke	Whether a stroke occurred in the patient	0 (no), 1 (yes)

#### 3.1.2 Dataset 2.

The 2,501 CT scans in the Brain Stroke CT Image Dataset, which was obtained via Kaggle, are divided into two groups: 1, 551 of the images depicted normal neurological characteristics and 950 images depicted characteristics of a brain stroke. ‘https://www.kaggle.com/datasets/afridirahman/brain-stroke-ct-image-dataset’. The given dataset aids in the development and enhancement of machine learning models for the purpose of accurately identifying stroke diseases from CT scans. As it can observe the details of the specimen’s brain structures and any relations to stroke, each picture within the morgue is critical. By using such information, the researchers and healthcare professionals can help make the approaches used by the machines more accurate and effective in identification of stroke, and, therefore, ensure timely and efficient treatment. Consequently, the given preparation is extensive it includes both typical and stroke images for model learning, which is helpful to enhance the framework, finally, the development of medical imaging and stroke diagnosis will be improved.

#### 3.1.3 Dataset concatenation – Multimodal dataset.

The databases for each site were concatenated by pairing each patient’s static features from Dataset 1 with their static imaging data from Dataset 2 based on their shared unique id. The approach is to merge age, gender and medical history, and lifestyle factors with CT scan images so that similar characteristics are concatenated with the CT scan data for analysis. Combining Dataset 1 and Dataset 2 will construct the new dataset that consider static and dynamic characteristics for stroke risk prediction. Dataset 1 encompasses demographic characteristics including individual identification numbers, gender, age, hypertension, heart disease status, marriage status, employment, type of dwelling, average blood sugar, body mass index (BMI), and smoking habits; the goal is the incidence of stroke. Dataset 2 contains 2,501 CT scan images, where 1,551 of them belong to normal neurological conditions; and 950 belong to stroke-affected conditions, which deliver multi-angled real visuals that seem quite significant in the diagnostic of the stroke. As a result, static features of Dataset 1 and static imaging data of Dataset 2 can be properly used in a proposed framework to establish the easier, comprehensive, and precise model for predicting the risk of stroke based on the fusion of data combining both types of data under the condition of protecting data privacy and security.

First, the appropriate datasets from the Dataset 1 and the Dataset 2 are collected and parsed in order to check for its data accuracy and relevancy. Data quality issues in which some of the values are missing in the dataset is managed using the mean and median imputation to ensure that no data is lost. Further, some attributes like those that age, the mean level of glucose, and BMI etc. are required to be normalized as they have higher values the effect will be huge, so Min-Max scaling is used. Since several classes may have more instances than other classes in Dataset 2 specifically, the SMOTE oversampling technique is applied to give more samples for the class that has few instances. After pre-processing, the datasets are merged by the patient ID on which the dependency of patient medical history, lifestyle variables, and CT image data is done. This integration of multiple data types allows the construction of an AI model that analyses one’s risk of having a stroke based on inputs from both the electronic health record and from the CT scan images. This generates a multimodal database of patient’s health that in turn increases the fidelity of stroke risk prediction based on comprehensive patient profiles and machine learning algorithms. Joining Dataset 1 with Dataset 2 to form the multimodal dataset entail merging the attributes within the datasets and stressing on compatibility when using the datasets. Each dataset contributes unique information: Dataset 1 contains demographic, medical, and lifestyle data, which are related to the likelihood of stroke prevention and Dataset 2, contains CT scan images of normal and stroke brain. To combine the datasets, it is necessary to use a key variable, for example, patient identifier, which will be used to pair the similar records in the two datasets. This connection allows the relation of patients’ characteristics to their corresponding CT scan images and the development of a comprehensive multimodal patient cohort, inclusive of clinical and imaging data. Combination of these complementary data sources is the detail of the multimodal dataset, which can supply the concrete ground for the AI model, and make more realistic and clinical journal’s prediction of stroke risk

**Fig 2:**
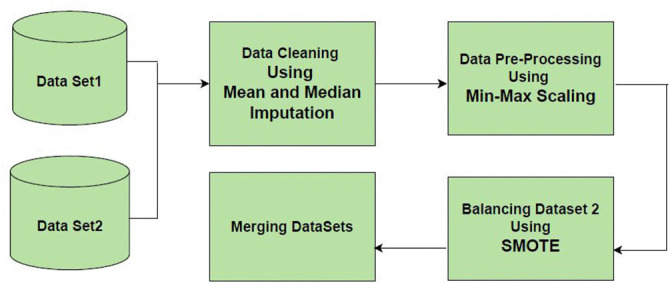
Formation of multimodal dataset for stroke risk prediction.

Fig 2 illustrates the process that is followed when developing multimodal dataset for stroke risk prediction.like Data Cleaning using Mean and Median imputation,Data Pre-Processing using Min-Max Scaling,SMOTE technique for balancing Dataset 2,and Merging DataSets.

### 3.2 Pre-processing using min-max normalization

One of the methods for making values comparable across distinctive characteristics is standardizing numerical characteristics and bringing them to a common range, often 0–1. This process is called min-max normalization. It gets even more critical when the features are of different sizes, as this step determines the optimum gains in the outcomes of the machine learning systems. The formula represented by Equation (1) is the Min-Max normalization.


$$C_{normalization}=\frac{C-C_{min}}{C_{max}-C_{min}}$$ (1)

Here, C_min and C_max are the feature’s minimum and maximum values in the dataset, respectively, whereas C is the original feature value.

In the Min-Max normalization method, it is mandatory to search all the numerical characteristic in max-min of the specific dataset. All feature values within the given range are scaled proportionately using the said extreme feature values as the reference. The normalizing formula acts on each of the values of the given features: if normalizing focuses on a feature, the formula is taken for each value of that feature and applied to it: each of the given to focus on features’ values is divided by the total sum of values after subtraction of the minimum value of the feature. Here, it is guaranteed that annotated numbers are between 0 and 1 where 1 is the most important while 0 remains least important. Finally, pursuing pre-processing of multimodal data for the prediction of the risk of stroke, numerical characteristics For example, the Min-Max normalization technique is used to age, BMI, and average glucose level. The study demonstrates with Dataset 1’s “age” feature as the example. Let us suppose that the minimum age among the individuals targeted in this dataset is 20 years and the maximum age 80 years. By subtracting minimum age of 20 from all the initial age values, and then dividing it with the range of the age values (80−20 = 60), the formula is then applied on each of the age values to get the age values equalized using Min-Max normalization. This procedure ensures that each age number is transformed to a standard scale of 0–1 this may assist in giving a fair comparison of data between age sets. In the same way, other numerical attributes in the given dataset go through this normalization process so that the necessary attributes are standardized concerning their ranges.

## 4. Stroke risk prediction utilizing federated MLP-GRU

### 4.1 Multilayer Perceptron (MLP)

A type of artificial neural network known as an MLP is composed of multiple layers of interconnected neurons, each of which has an input layer, several hidden layers, as well as an output layer. MLP is a powerful and versatile deep learning model that is widely used for a range of applications, such as recognition of patterns, regression, and classification. Neural units are arranged in layers inside an MLP, and all neurons are linked to all other neurons in the same layer. After entering the network through the hidden layers, the input layer gets the input information, which is subsequently used to generate estimates of output in the output layer. Equation (2) expresses a buried layer neuron’s output mathematically.


$$\overset{\left(l\right)}{\underset{j}{a}}=f\left(\sum_{i=\text{1}}^{n}\overset{\left(l\right)}{\underset{ij}{w}}\cdot \overset{\left(l-\text{1}\right)}{\underset{i}{a}}+\overset{\left(l\right)}{\underset{j}{b}}\right)$$ (2)

Where, $\overset{\left(l\right)}{\underset{ij}{w}}$ is the weight linking neuron i in layer l-1 to neuron j in layer l, $\overset{\left(l\right)}{\underset{j}{a}}$ is the stimulation of neuron j in layers l, f is the function of stimulation applied to the weighted sum of inputs, and $\overset{\left(l-\text{1}\right)}{\underset{i}{a}}$ is the activation of neuron i in the preceding layer. The bias parameter for neuron j in layer l is$\overset{\left(l\right)}{\underset{j}{b}}$. Complex nonlinear functions may be approximated by MLP thanks to the activation function f, which adds nonlinearity to the network. Sigmoid, tanh, and ReLU are popular activation functions; each has benefits and traits of its own. In machine learning training, backpropagation as a technique that enables the network to learn optimal weights and biases via propagating errors between expected and actual outputs backwards through the network and adjusting the weights to reduce these errors. Gradient descent methods, such Adam or stochastic gradient descent, are commonly used for this kind of optimization. They operate by iteratively modifying the parameters in accordance with the parameters’ reference to the gradients in the loss function.

As per the requirements of given study, an MLP may use a number of loss functions and mean squared and cross-entropy losses are used for regression and classification tasks respectively. The optimization process is leaden because the loss function the goal of which is to minimize prediction errors defines the type of optimization. Because of its capacity to recognize complex relationships and data patterns, MLP is used in a wide range of applications, from image as well as natural language analysis to financial forecasting for the detection of health issues. It is a crucial part of deep learning and drives progress in machine learning and artificial intelligence studies and implementations due to the network’s flexibility and powerful learning capacities. For input images in the federated MLP-GRU model for forecasting stroke risk, the images have a particular size suitable for the neural network to handle. How the kernels used is defines the spatial resolution required in feature extraction. The stride applied (Stride 1 or 2) affects feature maps down sampling and allows regulating the size of the feature maps. The activation function applied in this study is generally ReLU because of its performance to introduce non-linearity and more efficiently learn deep architecture

### 4.2 Recurrent units

In order to tackle the problem of vanishing gradients which normal RNNs face recurrent neural network architectures of the GRU kind are designed. GRUs can describe long-term dependencies, at the same time avoiding certain issues resulting from vanishing gradients, which makes the model valuable for sequential data such as data history or natural language. The main components of a GRU are a sequence of update gates combined with reset gates, which control the data, pass through the network. Through these gates, GRUs are capable of retaining the information that is relevant for the interaction in the course of an extended sequence while being able to update and forget information at varying rates. The update gate $z_{t}$ and reset gate $r_{t}$ may be calculated mathematically using Equations (3) and (4).


$$z_{t}=\sigma \left(W_{z}\cdot \left[h_{t-\text{1}},x_{t}\right]+b_{z}\right)$$ (3)

$$r_{t}=\sigma \left(W_{r}\cdot \left[h_{t-\text{1}},x_{t}\right]+b_{r}\right)$$ (4)

Where, an input at the time level t is denoted by $x_{t}$. The concealed state from the preceding period of time is represented by$h_{t-\text{1}}$. The weight matrix for the updated and reset gates are denoted by $W_{z}$ and $W_{r}$, respectively. Bias vectors are $b_{z}$ and $b_{r}$. The sigmoid function for activation is σ. The amount of the prior hidden state $h_{t-\text{1}}$ to keep and the amount of the present input information and the current input is given in Equation (5).


$$\tilde{h_{t}}=tanh\left(W\cdot \left[r_{t}⨀h_{t-\text{1}},x_{t}\right]+b\right)$$ (5)

Where, the symbol ⨀ stands for multiplication of elements. The weighted matrix for the potential concealed state is denoted by W. There is a bias vector, b. The hyperbolic tangent function of activation is denoted by tanh. Ultimately, the true concealed state $h_{t}$ is calculated by employing the updated gate $z_{t}$ to interpolate between the individual concealed state $\tilde{h_{t}}$ and the prior concealed state $h_{t-\text{1}}$ is given in Equation (6).


$$h_{t}=\left(\text{1}-z_{t}\right)⨀h_{t-\text{1}}+z_{t}⨀\tilde{h_{t}}$$ (6)

Recursive gradient computation through unfolded steps in time is how GRUs are trained; throughout training, the neural network finds out ideal biases and weights in order that reduce an established function of loss for categorization tasks. GRUs are a form of RNN design which tackles the difficulties of modeling sequential information through the use of update and reset gates to specifically update and forget information over time. This mechanism enables GRUs to capture dependency relationships in sequential information effectively. These gates allow GRUs to effectively record long-term dependencies in data, which makes them ideal for applications like speech recognition, natural language interpreting, and time series forecasting.

### 4.3 Hybrid MLP-GRU

To enhance the accuracy of risk forecasts, the stroke risk prediction model uses the benefits of both MLPs and GRUs to create the MLP-GRU model. Analyzing the results, it should be mentioned that the GRU component is more effective in dealing with sequential data, for example, time series of clinical parameters, while the MLP component fits better for dealing with static features, which include age, gender, medical history, and lifestyle factors. By combining both designs, the hybrid model is able to offer a more accurate measure of the patient’s risk of stroke due to the ability to calculate static and dynamic factors and accommodate the complex interrelations between the two. In this way, meaningful representations are obtained from features applying the MLP on static characteristics while dealing with the dynamic ones. After the concatenation of these properties, through the temporal dimension, the sequential data is then passed through several GRU layers in order to extract temporal cycles and relations. To develop the stroke risk prediction, the results generated in the two elements are passed into a final dense layer. This integration improves the model’s robustness and accuracy while determining the risk of stroke by incorporating the contextual data combined from the sequential pattern and the discriminative static characteristics.

### 4.4 Federated MLP-GRU for stroke risk prediction

**Fig 3:**
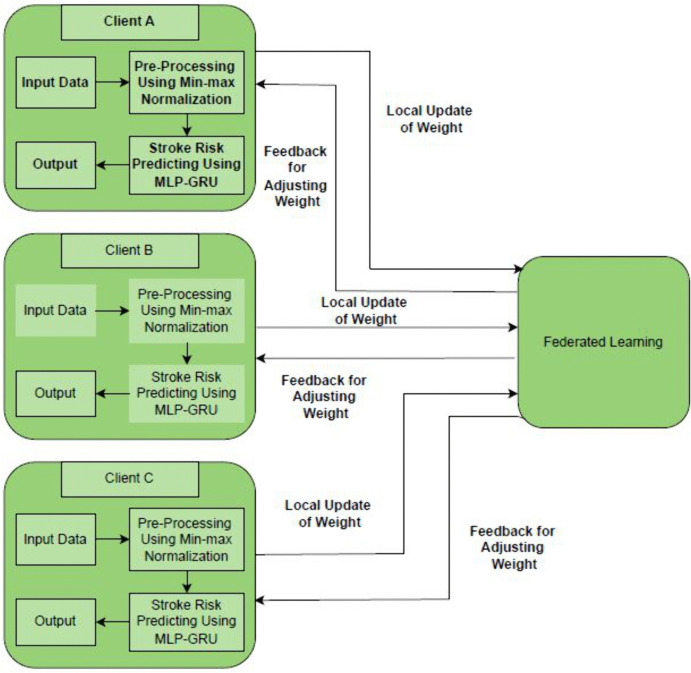
Federated MLP-GRU architecture for stroke risk prediction.

The Federated MLP-GRU model used for stroke risk prediction extracts the factors of several datasets through federated learning to improve data privacy, security, and communication of entities with different data. Within this framework, there are three clients namely: Client A, Client B and Client C, which are used to perform various steps in relation to the data. The client feeds the multimodal dataset to be processed from where it is split into three equal parts. In more detail, the dataset incorporates both the spatial and the temporal characteristics of the stroke patients, which is the main advantage of the dataset. The Federated MLP-GRU for Stroke Risk Prediction can be depicted as displayed in the Fig 3.

The Federated MLP-GRU model is developed to classify the risk of stroke by combining static and sequential data in a single model while protecting patient information by using federated learning. The demographic, medical and lifestyle characteristics are passed through a MLP that converts these features into a discriminant feature space. At the same time, sequential data, present as the sample order of clinical time-series parameters or imaging time-series, which are inputted to the GRUs. Last, the MLP and GRU are then concatenated and processed through a dense layer to predict the stroke risk. This hybrid architecture increases the efficiency of the prediction by including both static and temporal dependencies within the data.

### 4.5 Federated Learning Integration

When it comes to privacy regulation, the federated learning framework acts as a guard that only trains the MLP-GRU models on several clients. Every client analyses its portion of the multimodal dataset representing static and sequential modalities. Each client then sends updates to the a centralized server for its models (such as weights and bias) during local training. The global model in equation (7) is calculated by the server by aggregating these changes using Federated Averaging (FedAvg):


$$\theta_{global}=\frac{\text{1}}{K}\sum_{k=\text{1}}^{K}\theta_{k}$$ (7)

where K is the number of clients, and $\theta_{k}$ constitutes the model parameters from client k. The improved global model is sent back to the clients to learn further in the local model and this forms a loop. This approach minimizes data leakage since raw patient data are never transferred to the cloud yet, multiple datasets are used for better generalization. Overcoming the flaws of MLP and GRU, the proposed work integrates both MLP for static features and GRU for sequential pattern extraction to accurately and stably predict stroke risks.

Here, in this Fig 3 federated learning architecture with 3 different clients, all the clients will train their MLP-GRU model locally on the clients’ dataset. The clients subdivides the multimodal dataset into three equal parts through which they process it. The proposed multimodal dataset encodes the interactions between the static and sequential features for an effective stroke risk assessment. Once the local training is completed, each of the clients transmits its new model to a central server. These updates are collected and summarized by the server, which in effect generates a new model of the fed learning for global without sharing the actual data, thus keeping the data private. The weights of this model are then sent back to the centralized server where it is then distributed back to the clients to offer feedback to update their weights. The general iterative training and global averaging of the several newly trained local models go on until this model is obtained, which forms a powerful and reliable stroke risk prediction model. This considerably improves the predictive ability in the federated learning system whilst improving privacy and security of data by implementing MLP for static data and GRU for sequential data. The federated MLP-GRU integrated the various datasets collected from various sources by the various health centres and thus improved cooperation among them while also improving on the precision and personalized assessment of risks of stroke. Besides, it increases the accuracy and stability of the model as well as solves ethical and legal problems of data sharing.

**Algorithm: Federated MLP-GRU Stroke Risk Prediction**

Input: Dataset 1: Demographic, medical, and lifestyle factors, Dataset 2: CT scan images (normal and stroke-affected conditions).

Output: A federated learning-based global model that predicts the risk of stroke.

Step 1: Collect Dataset 1 and Dataset 2 from Kaggle, handle missing values using imputation, normalize numerical features using Min-Max scaling, address class imbalance in Dataset 2 using the SMOTE technique, and create a multimodal dataset by aligning each patient’s static features from Dataset 1 with their corresponding imaging data from Dataset 2 using a common unique identifier.

Step 2: Deploy three clients (Client A, Client B, and Client C), where each client processes the multimodal dataset by splitting it into three equal parts. The multimodal dataset capture the complex interactions between static and sequential features for a comprehensive stroke risk prediction.

Step 3: Each client trains its respective MLP-GRU model locally on its dataset.

Step 4: Static characteristics of Dataset 1 are processed using an MLP. Following the combination of these features, sequential data of Dataset 2 are processed through several GRU layers to identify patterns and temporal relationships. The outputs from these two elements are then combined and fed into a final dense layer to create the stroke risk prediction.

Step 5: Each client sends its local model updates to a centralized server without transferring raw data.

Step 6: To generate a global model, the centralized server uses Federated Averaging to aggregate the local model changes.

Step 7: For additional training, the local clients receive the aggregate global model from the centralized server.

Step 8: Repeat Steps 3 to 6 iteratively until the model converges, resulting in an enhanced and comprehensive stroke risk prediction model.

Step 9: Deploy the final global model for stroke risk prediction, ensuring accurate, privacy-preserving, and comprehensive assessments leveraging both static and sequential data.

The integration of MLP and GRU in federated learning increases the efficiency of the stroke risk prediction models based on their advantages. The MLP efficiently deals with the static features forming input data matrices, including demographic and medical history; at the same time, it can describe the complex feature interactions, such as age, gender, and lifestyle, between feature vectors and capture both simple and complex feature relations. On the other hand, dynamic data such as patients health records over time, and sequential data where the GRU excels at capturing long-term dependencies and temporal sequences. In combination with the prediction of transient characteristics, the static and dynamic models complement one another for the assessment of the stroke risk and generalize better compared to the single model predictive evaluation.

## 5. Results and discussion

The proposed stroke risk prediction methodology uses a federation learning structure in which MLP and GRU networks are combined to use multiple datasets while preserving privacy.

### 5.1 Implementation platform

The proposed federated MLP-GRU model for stroke risk prediction involves computing platforms like python used to learn the model that operates optimally with deep learning architectures particularly when operating in federated environments. This setup involves deployment of computing resources in different clients to warrant the privacy of the data collected. Data is locally trained on each PDA, while the federated learning model is performed on cloud-based platforms that support the specific framework. Appropriate techniques are incorporated through machine learning platforms like TensorFlow, PyTorch or other particularly developed federated learning frameworks like TensorFlow Federated or PySyft in order to train local models as well as to accumulate them at a superior server. This platform is suitable for the processing of a large number of cases and inclusion of the multifactorial data characteristics, such as demographic and clinical data, and MRI, which are crucial to estimating stroke risk accurately. The other attribute that makes the use of federated learning to reduce the challenges of data privacy is that the raw data does not have to move between the clients and the central server.

### 5.2 Dataset details

Dataset 1 contains patient’s demographics, medical and living history, which is used to diagnose and monitor the patients in question and Dataset 2 contains CT images of the patients. Among these datasets some possess missing values and for such datasets, imputation is done,normalization is performed through Min-Max scaling the SMOTE technique used will handle class imbalance. Every client receives three local models to fine-tune on and these include Client A, Client B and Client C. The clients also partition the multimodal dataset into three equivalent sets for their processes. The input vector features of this dataset are equals the independent and dependent ones as well as the sequence ones important for the stroke risk prediction.

The Local model (MLP-GRU) of each client updates their model’s parameters, sends them to a central server, and apply Federated Averaging to boost the global model. Static attributes of Dataset 1 are fed to an MLP. Subsequently, the features mentioned above blend together and, therefore, sequentially ordered data of Dataset 2 are passed through more GRU layers to learn the patterns and temporal dependencies. The results from these two elements are then joined and passed through a final fully connected layer to produce the stroke risk forecast. It promotes the comprehensive, accurate, and privacy-preserving model-of-stroke risk prediction collaboratively through the common connection of healthcare systems for successful early and individualized assessment of stroke risk.

#### On unique IDs across datasets.

Clinical and imaging data were integrated using anonymised patient IDs from each dataset samples. Clinical feature identifiers were checked for agreement with CT scan identifiers in process. Aligning metadata parameters including age, sex, and acquisition timestamps confirmed the join in circumstances when the dataset portal did not explicitly declare this correspondence. This method correctly integrated a patient’s clinical records and imaging data into a single representation, enabling accurate and reliable multimodal fusion.

#### Selecting clinical and imaging data models.

Different models were used for the two modalities to account for their differences. A gradient boosting classifier was used for clinical tabular data due to its tolerance to structured inputs and non-linear feature interactions. Given its success in extracting spatial hierarchies from medical pictures, a convolutional neural network (CNN) was used for imaging data. By training these models separately on their modalities, complimentary feature embeddings were obtained and merged into a multimodal fusion framework. This modular approach modeled imaging and clinical data streams using methods that fit their properties.

#### Choosing GRU for image processing.

The imaging process used gated recurrent units (GRUs) as a supplement to convolutional layers. CNNs extracted main features from CT images, and GRUs enhanced convolutional layers’ sequential representation. The necessity to record temporal coherence and contextual relationships across volumetric slices prompted this approach. Local spatial properties were preserved when modeling inter-slice continuity by embedding recurrent units after convolutional stages, which is important for thoracic CT datasets where illness patterns may span multiple slices.

#### The nature of federated learning.

The experimental framework simulates federated learning by partitioning the same dataset among different clients to emulate distributed data conditions. This method managed federated optimization algorithm testing and ensured consistency. The data source was single, but the partitioning technique mimicked heterogeneity in real-world federated healthcare systems, where clients have overlapping but non-identical subsets of information sets.Distinguishing it from real-world federated installations involving geographically scattered hospitals or institutions in process.

#### Dataset characteristics and preprocessing.

Each of the 2,000 patient records included structured clinical factors and volumetric CT imaging. Imaging data was axial CT scans pre-processed to consistent voxel spacing, whereas clinical variables included demographics, laboratory results, and comorbidities. Normalizing continuous clinical variables, encoding categorical values, and impute missing data using mean or mode were preprocessing stages. Imaging scans were resampled to consistent resolution, cropped to the segmented region using automatic segmentation, and intensity-normalized to standardize Hounsfield unit ranges. These methods harmonised data across modalities and made experimental workflow sets reproducible sets.

**Fig 4:**
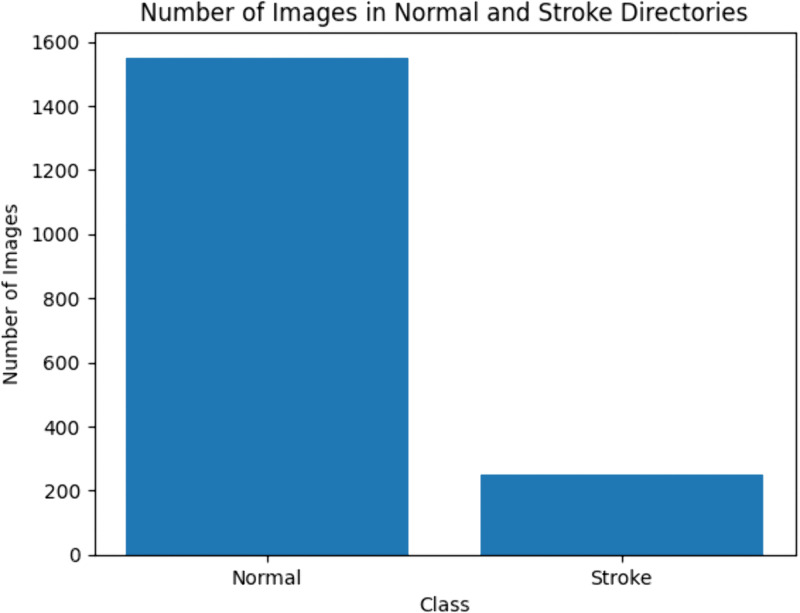
Comparison of the number of individuals with and without stroke.

The amount of images in two directories: one for normal neurological conditions and the second for conditions affected by stroke was provided in the form of a comparison bar chart as depicted in the Fig 4. On the x-axis, Normal, Stroke is given as the title and on the Y-axis, it places the number of images. Inability to observe class unbalance within a dataset is made easier to observe with this image, as it is vital in machine learning model training. An imbalance may lead to an undesirable situation affecting the accuracy of the model and it may come up with biased decision. An example of an imbalance would be having a larger number of standard images than stroke images, or having far fewer of each. The figure underlines the necessity of such methods as SMOTE to ensure the balance while training models, with the main target of improving the accuracy and reliability of the stroke recognition algorithms. The first facet or phase of losing data in modelling is pertinent to understanding the structure of the dataset as well as guidelines for future phases in the model generation.

**Fig 5:**
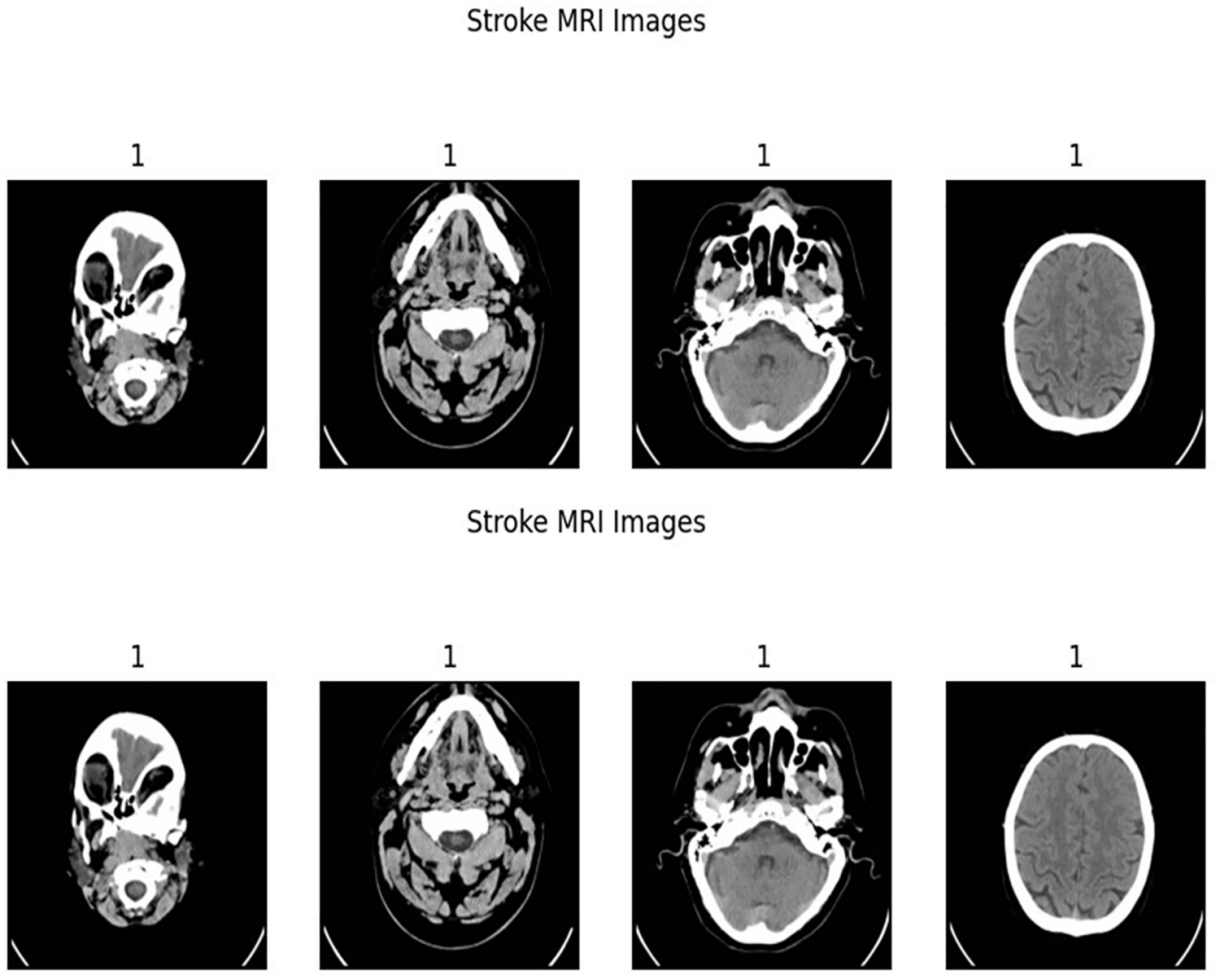
Reading, resizing, normalizing, and visualizing images the MRI images.

Fig 5 depicts MRI images of neurological states that are normal and neurological states that differ because of the occurrence of the stroke. Before feeding them to the neural network, the photos were resized to the form of 128 by 128 pixels and pixel intensity value was scaled to the range of [0, 1]. The set of photos with examples from the “Normal” group is presented in the top horizontal row, and the “Stroke” group is presented in the bottom row with labels 0 for ‘Normal’ and 1 for ‘Stroke’. This depiction is beneficial when establishing and training the machine learning algorithms for the future detection of stroke as it allows the subject to easily and efficiently discern the visual differences between the two categories. From these examples, it can be seen that the data concerning images are complex and diverse, which requires having powerful algorithms that would help find traces of stroke in minor details. Moreover, the side-by-side breakdown reveals how resizing and normalization pre-processing steps are inevitable for an identification of equal dimensions of input and scaling features that are crucial in training a model and getting accurate predictions. This visual validation initiates the process of checking the quality and suitability of the image data which is stored in the database to be used in later analytical and predictive modelling processes.

**Fig 6:**
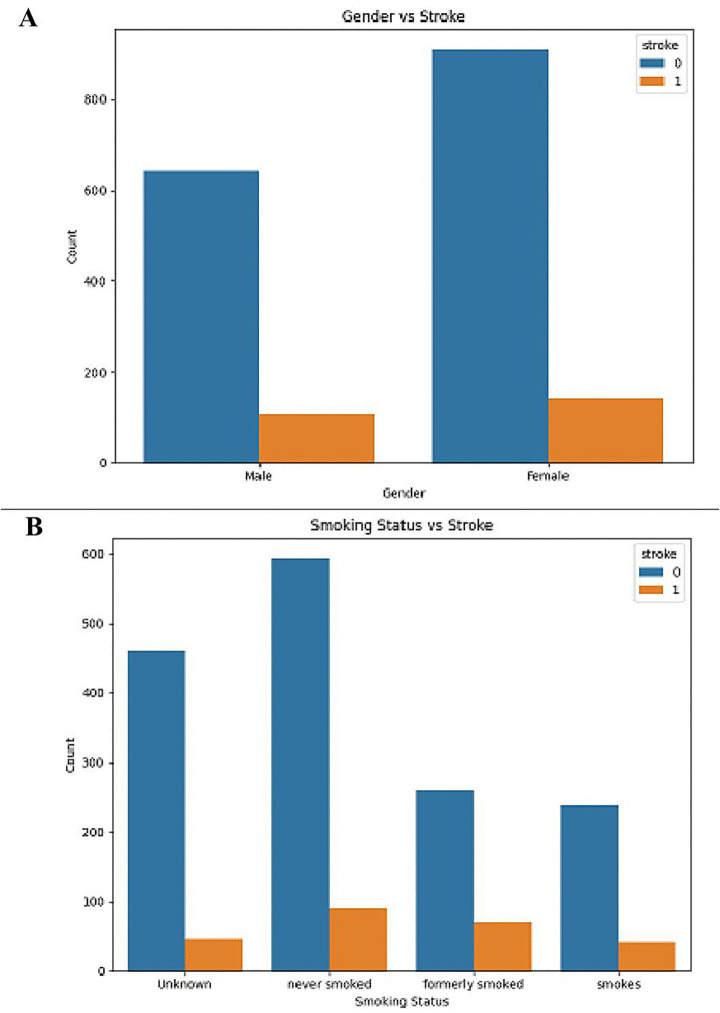
Comparison of Stroke Between (A) Gender vs stroke (B) Smoking Status vs Stroke.

The association between gender, smoking status and occurrence of stroke is depicted the two bar plots in Fig 6. The first plot (A)shows the proportion of the stroke incidences by gender where the gender field has been classified into male, female and other. The bars are split in the ratio correlating to the portion of stroke victims among males or females. This figure demonstrates a way of presenting probable gender disparities in regard to stroke by establishing whether there are significant differences in the occurrence of stroke in either male or female people. The second plot (B) deals with the effect of smoking status on the occurrence of the stroke. Individuals are categorized into four groups according to their smoking habits: other, did not smoke, and smoked in the past and current smokers. Chains are divided to display the rates of strokes in each smoking status. This makes it possible to examine the hypothesis that smoking increases the risk of the stroke as well as examine and test the smoking-smoking behaviours relationship. However, when all these ideas are summed up, the reader gains a satisfactory understanding of the possible relations between smoking and gender regarding strokes. They also assist in determining population groups that are vulnerable with an aim of providing them with enhanced preventative measures. In the end, they are made flexible and easy to comprehend by the employment of sub plots and proper labelling which also points out certain trends and future research sectors.

**Fig 7:**
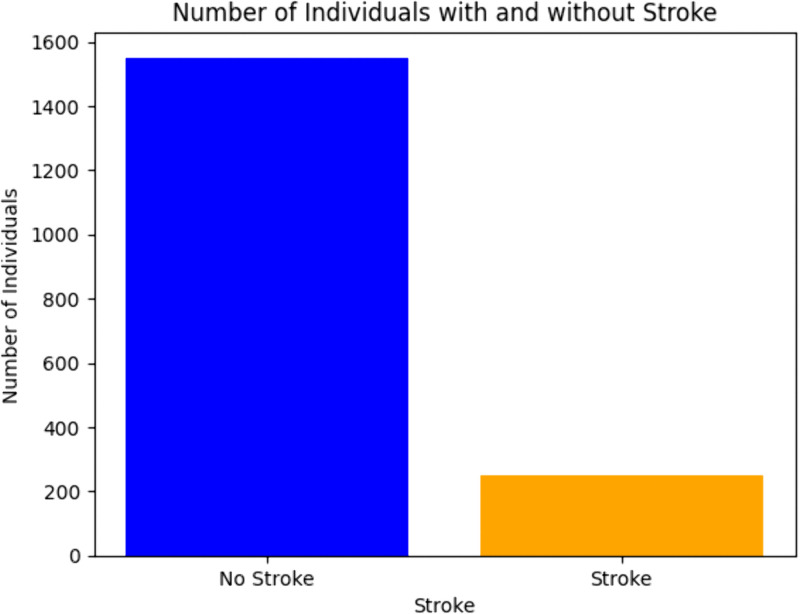
Number of individuals with and without stroke.

The frequency distribution of the stroke cases is illustrated in Fig 7 in the form of a bar chart. The x-axis represents two categories: of the respondents grouped under Non-Stoke where they indicate they have never had a stroke in their lifetime and the Strokes where they have had one sometime in their lifetime. The number of individuals in each group is counted on the y-axis. The number of persons who had no strokes was indicated by the blue bar, while the total number of people who had strokes was indicated by the orange bar. It is clear from the plot that there are a lot more people who have never had a stroke than who have, suggesting that the dataset is unbalanced and that most of the instances are not strokes. This imbalance is crucial to recognize as it can influence the performance of predictive models, potentially leading to biased outcomes if not properly addressed during the data preprocessing and model training phases. The obvious variation in bar heights emphasizes the necessity of methods like oversampling, undersampling, or using appropriate evaluation metrics to ensure the model effectively learns from both classes.

**Fig 8:**
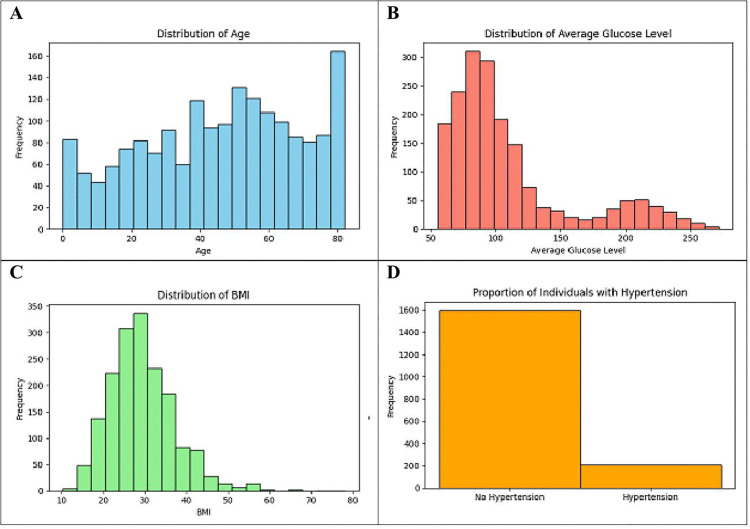
Comparative analysis between (A) Age, (B) Glucose, (C) BMI, and (D) Hypertension Distribution among Individuals.

Fig 8 presents a comprehensive analysis of key health metrics among individuals in the dataset through a series of histograms. In the first subplot (A), the distribution of age is depicted, showing a broad range of ages with a higher frequency of individuals in the middle age brackets. The second subplot (B) illustrates the distribution of average glucose levels, which indicates a right-skewed distribution with most individuals having glucose levels clustered around the lower end, but a significant number also displaying elevated levels. The third subplot (C) shows the distribution of BMI, which is slightly right-skewed, suggesting a majority of individuals have a BMI within the normal to overweight range, but also a notable proportion with higher BMI values. The final subplot (D) represents the proportion of individuals with and without hypertension using a bar plot. The majority of individuals do not have hypertension, but there is a substantial number of cases with hypertension. These visualizations provide essential insights into the demographic and health-related characteristics of the dataset, highlighting the diversity and common health issues such as high glucose levels and hypertension, which are crucial for understanding and predicting stroke risk.

**Fig 9:**
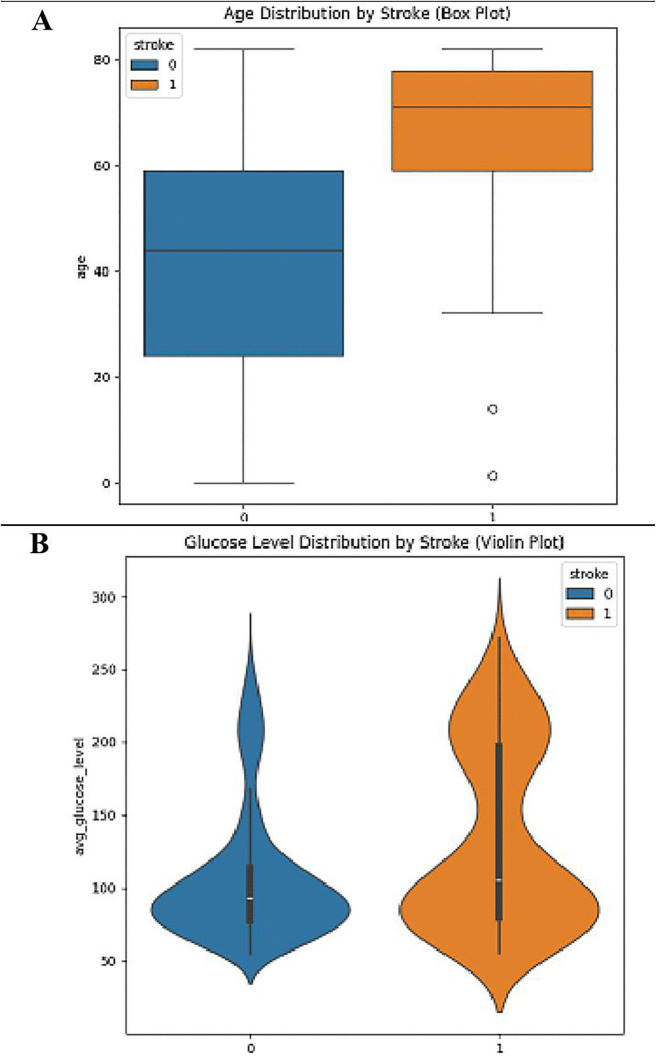
Relationship between(A) Age, (B) Glucose Level, and Stroke Occurrence.

Fig 9 presents a combined analysis of the relationship between age, glucose level, and stroke occurrence through two distinct plots: a distillation of ages in form of a box plot and an illustration of glucose level using violin plot, both with respect to stroke individuals. The first subplot (A) presents a box plot of age in regard to stroke and no stroke has shown the age of subjects who underwent a stroke as higher, as it has a higher median age and a wider IQR compared to the group of patients without a stroke. This implies that there is a direct relationship between stroke incidence and age as well, more strength. The distribution of average blood glucose levels in individuals both before and after a stroke is shown in the violin plot (B) on the right. For one it points out that precedent stroke patients bear higher prevalence of hyperglycemia and the histogram shows a wider and a higher rise of the stroke group’s tails. This plot contains features of a box plot and a kernel density plot, so it gives a full picture of the navigators’ glucose levels density and dispersion. Altogether, these graphs assert that both age and blood sugar levels are critical indicators that predispose one to stroke, asserting the need to monitor both parameters for stroke prevention.

**Fig 10:**
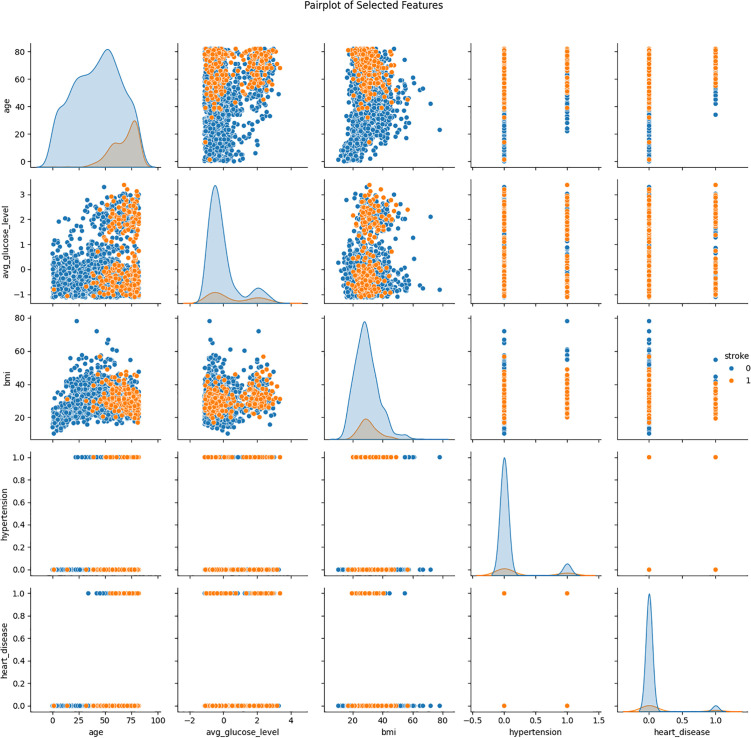
Pairplot of Heart Disease, Stroke, Hypertension, BMI, Average Glucose Level, and Age.

Fig 10 presents a Pairplot of Heart Disease, Stroke, Hypertension, BMI, Average Glucose Level, and Agewhere pairwise comparisons of these features are presented as colored by the models’ stroke status. Every subplot in the matrix enables the researcher to examine the relationship between two variables and diagonal plots show the distribution of individual features. For instance, where the age against average glucose plot indicates possible clustering by using striation, the use of stroke and the power of such signs may indicate more general clustering; those who had a stroke will naturally have larger glucose levels and will generally be older. In the same way, the involvement of hypertension and heart disease also suggest that such ailments seem to be more recurrent among stroke patients from the way of plotting data that is scattered and displayed by different density. The most critical and overarching aspects reveal that the stroke patients are generally older, their glucose level is higher as well as their BMI, and they are more significantly prone to hypertensive and ischemic heart diseases. Therefore, contrasting different hues for stroke and non-stroke individuals enables presenting these distinctions vividly and in addition to that; this approach helps understand coordination between health metrics and strokes in order to assess the predictors and risk factors of strokes.

#### Novelty of the analytical method process.

Layered merging of CNN-based imaging representations with gradient boosting on clinical characteristics, harmonised by recurrent modelling, makes the proposed framework innovative. The multi-level integration captures intra-modality and cross-modality relationships, distinguishing it from unimodal or shallow multimodal methods. The system also bridges laboratory studies and privacy-sensitive real-world deployments with a simulated federated context, contributing to machine learning research and medical informatics in process.

**Fig 11:**
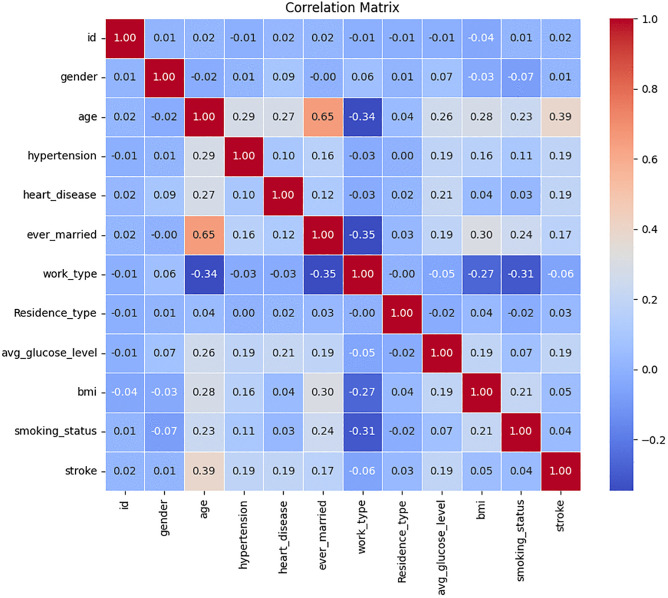
Correlation matrix of health parameters and stroke risk factors.

Fig 11 illustrates the correlation matrix heatmap that evaluates the correlation between various medical parameters of the dataset and the factors related to stroke. In the heatmap, each cell represents the correlation value of two variables, the correlation value range between −1 and 1. Positive coefficients refer to a direct association where an augmentation in one variable tends to result in augmentation of the other, negative values point at an inverted relationship whereby an improvement in the value of one variable corresponds to a decrease in the value of the other variable; coefficients close to zero suggest that, there is likely to be very little or no direct linear relationship. This concluded that age and heart disease or hypertension have a closer relationship, implying that elderly people are more susceptible to acquire these conditions. Additionally, there is a positive correlation between the occurrence of stroke and heart diseases or hypertension; hence, patients with such diseases have a high chance of suffering from a stroke. Such relationships are described in detail and visualized nicely in a heatmap, providing a summary of the interrelations between different health indicators and the risk of stroke. Such knowledge is useful in defining critical risk factors and possibly the areas for further research and medical treatment.

**Fig 12:**
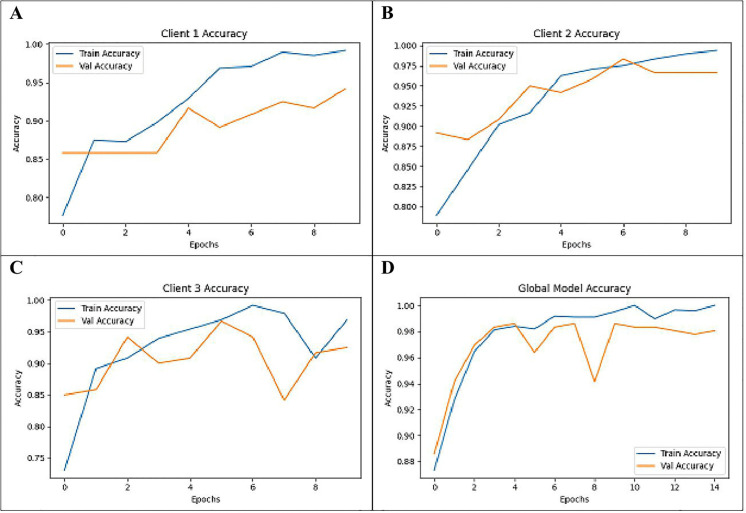
Training and Validation Accuracy of (A) Client 1, (B) Client 2, (C) Client 3 and (D) Global Model.

Fig 12 shows the Training as well as Validation Accuracy metrics along with the training performance of the four clients (Global Model, Client 1, Client 2, and Client 3) within the FL framework. The graphs capture the accuracy dynamics in the federated learning setup for three clients and the global model. Nearly 99% training accuracy alongside nearly 93% validation accuracy were attained by the first client shown in graph (A); 100% training accuracy along with almost 97% validation accuracy were attained by the second client Shown in graph (B); and nearly 99% training accuracy as well as nearly 92–93% validation accuracy were attained by the third client shown in graph (C). The global model attains 99% for training and 98% for validation when converge shown in graph (D). The hyper parameters are: learning rate = 0.001(with decay strategy if available), Local client training and global aggregation batch size = 32, no. of epochs per client = 10, the Adam optimizer, and cross entropy loss for fine tuning accuracy performance.

Every client optimizes its model on a local portion of the data, and this leads to individual training and validation accuracy that depicts the model’s performance in unique client’s environment. These accuracies are useful in determining the performance of the local models prior to the time when they are updated in the centralized server. The Global Model, which accumulates the updates from all clients by methods like Federated Averaging, displays its own Training and Validation Accuracy, which reflects the total sum of accuracy on data of all the clients. This global metric is significant as it measures the overall performance and effective transfer ability of the compounded model by integrating various types of data distributions from all the clients. Using these accuracies for both the clients and the Global Model, it is possible to estimate the degree of heterogeneity in the data, the benefits of federated learning approach, and further need to fine-tune the model aggregation/initiation processes or the local training methodology.In the federated learning setup, data heterogeneity across the three clients is well managed by training the MLP-GRU model of each client locally on their patient dataset since patient characteristics are diverse. These local models are updated, centroid or more accurate representations are calculated by the central server and then broadcasted to all the connected subunits to maintain model uniformity, whilst respecting data confidentiality and integrity.

**Fig 13:**
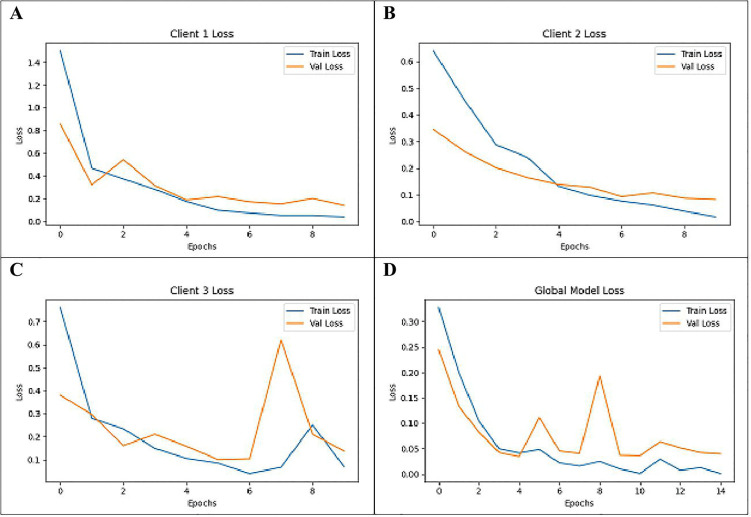
Training and Validation Loss of(A) Client 1, (B) Client 2, (C) Client 3 and (D) Global Model.

The Training and Validation Loss which have indices of Client1, Client2, Client3, and the Global Model are the key factors that determine performance a model in a federated learning setting is depicted in Fig 13. The graphs provide information on the loss trend during training and validation phases from three clients and the global model in applied federated learning. Client 1 shown in graph (A) has a training loss going down to a value close to 0.02 and a validation loss which remains close to 0.1. Client 2 shown in graph (B)gets nearly 0.01 training loss and roughly 0.05 validation loss out. From the training particulars we infer that Client 3 shown in graph (C) has a training loss reducing to approximately 0.05 with the validation loss varying to the maximum of 0.6 before returning to its steady state. The global model shown in graph (D) also is by approximately 0.01 in the training loss and around 0.05 in the validation loss. These results stress the most efficient loss reduction with the least changes during the global aggregation.

To note, these metrics demonstrate how the models are learning about the local client’s data as well as summarizing the global information. Therefore, each client, using its portion of the data set, goes through specific training and validation loss scores that estimate the error or misfit between the learning model’s output and the unavailability of real data throughout the local training phase. Such loss values are critical in determining the extent to which each client’s model is eliminating the error rate, where a lower value is desirable. After each client completes its training, the model updates are sent to a centralized server where the Global Model is formed through techniques like Federated Averaging. The Training and Validation Loss of the Global Model represents the overall error across the entire distributed dataset, encompassing the contributions from all clients. Comparing these loss values across individual clients and the Global Model helps in assessing the consistency of the training process, the diversity in data distributions among clients, and the success of the global model in reducing errors across the entire federated system. This comparison also highlights any potential issues with overfitting or underfitting, guiding further optimization of both local and global models.

**Fig 14:**
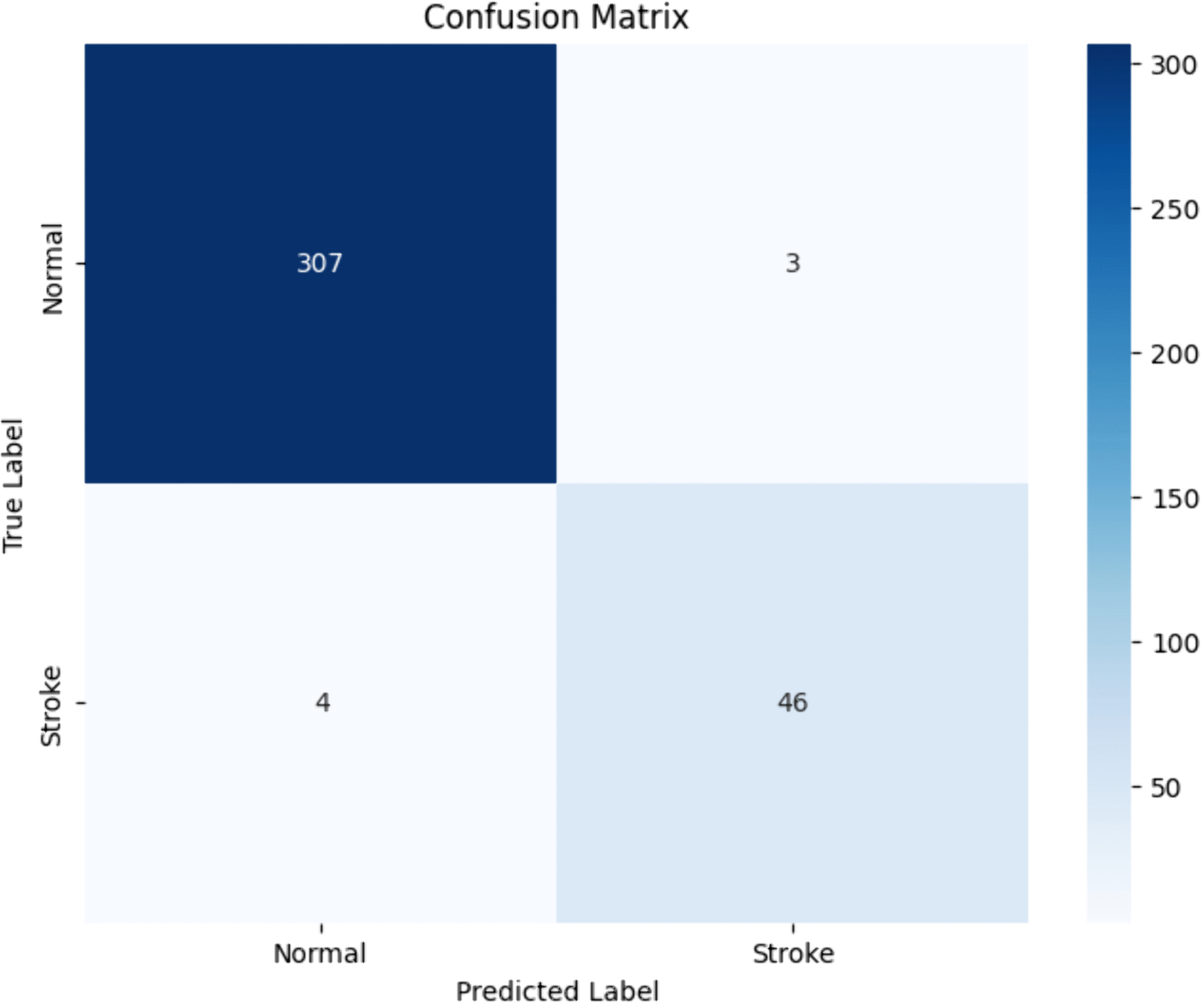
Confusion matrix for stroke prediction.

Fig 14 confusion matrix offers a thorough analysis of a binary categorization model’s performance, especially differentiating among “Normal” and “Stroke” situations. The genuine labels in this matrix are represented by the rows, while the anticipated labels are represented by the columns. In the matrix, the number of predictions that fit into each respective category is displayed in each cell. The top-right cell shows the total number of false positives, the top-left cell shows the total number of true negatives, the bottom-left cell shows the total number of false negatives, and the bottom-right cell shows the total number of true positives. While the elevated numbers in the off-diagonal cells point to places where the model is making mistakes, high values in the horizontal cells indicate strong model performance. Darker shades on the heatmap indicate more counts, giving a visual indication of the number of predictions. This visual aid facilitates the rapid evaluation of the model’s memory, accuracy, precision, and general capacity to discriminate between the two categories. The confusion matrix may be used to detect certain mistake kinds and to brainstorm ways to optimize the model’s performance, including rebalancing the dataset, changing the decision threshold, or refining feature selection.

**Fig 15:**
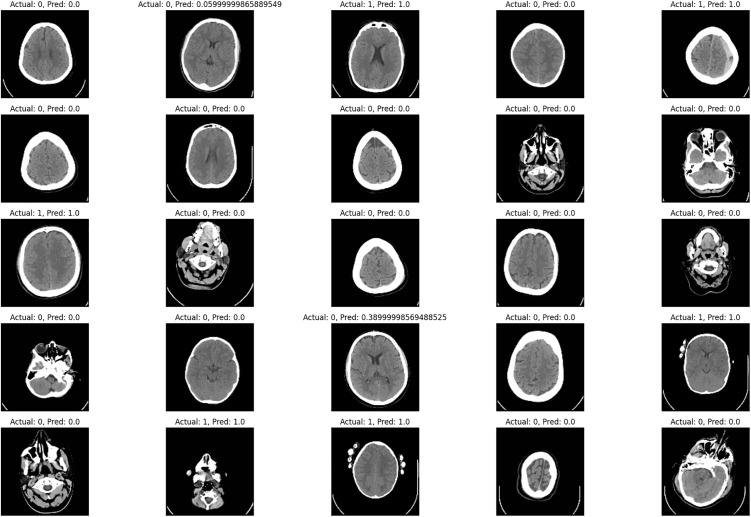
Actual vs predicted of the brain CT images.

Fig 15 displays a grid of 25 randomly selected images from the test set, each accompanied by its actual label and predicted value. Every subplot in the 5 by 5 grid displays an MRI image; the title means the ground truth, which is Actual, and the probability that the image belongs to the stroke group according to the model, which is Predicted. The actual labels vary, with Normal assigned as 0 and Stroke as 1, while the predicted values are numerical, usually between 0 and 1 that reflect the model’s certainty regarding the diagnosis of stroke. This makes it easy to assess the correspondence between the model’s predictions and the actual labels in an endeavour to determine how well the model fared on given cases. It helps find out cases where the predictor is good in its prediction as well as cases where the model might have some errors in its prediction thus enabling the amendment of the model’s accuracy. The images are well aligned with the actual and the predicted values labelled, so it becomes easy for one to determine the performance of the model in question visually.

**Fig 16:**
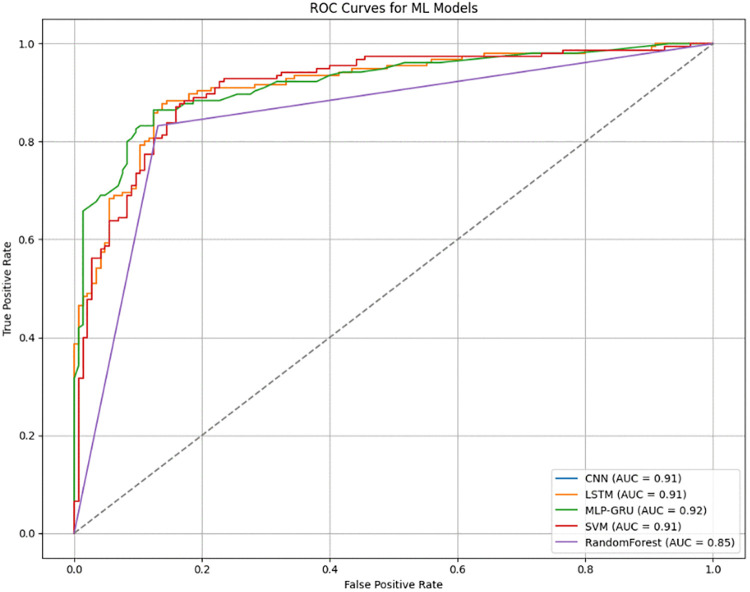
ROC curve for ML models.

An additional graphical representation which can be used to assess the performance of a binary classifier is a ROC curve, as shown in Fig 16. Plotting the True Positive Rate versus the False Positive Rate at different thresholds is what the curve does. The ratio to actual negative cases which have been mistakenly labeled as positive is measured by FPR(False positive rate), while the ratio for actual positive cases than the model correctly identifies is measured by TPR. The diagonal line indicates the accuracy rate of what is considered a naïve classifier or a model that randomly predicts. The performance of the model is better in terms of separating the positive and negative classes the closer the ROC curve gets to the left top quadrant. Higher accuracy in Region Better is depicted by a value of AUC(area under the ROC curve) closer to 1, a performance measure under the Receiving Operating Characteristic (ROC) Curve. This plot is useful to assess the model’s approach for categorization since it presents the ROC curve and AUC score of the model.

**Fig 17:**
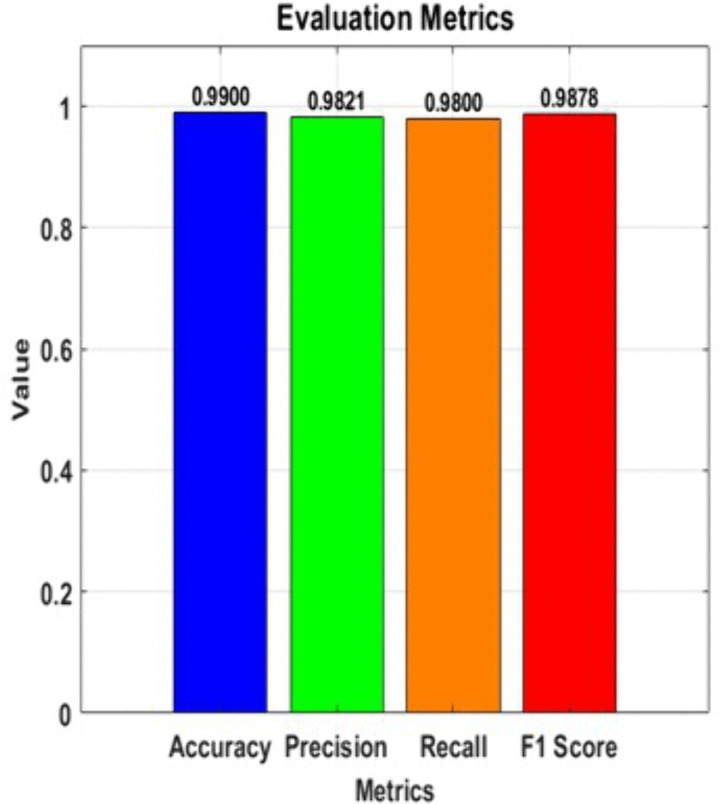
Evaluation metrics of the proposed method.

In light of this, the Assessment of the classification model is depicted graphically in Fig 17 below; therefore, the assessment metrics of the classification model include the following parameters; each bar the height of which is equivalent to the value of the given metric represents one of these metrics. Accuracy’s objective is to calculate the proportion of true findings among all the examples analyzed to assess model precision. The quality of the true positive projection out of all the positives is used to determine a model’s accuracy. As the proportion of true positives that the model accurately provides, sensitivity, also known as recall, gauges a model’s capacity to detect all pertinent cases. Unless very specific information is desired at the cost of certain inaccuracy, overall performance of a model is mentioned using a comprehensive metrics known as F1-Score, which is given as the harmonic mean of accuracy and recall. Achievement across these crucial assessing factors has been depicted in bars and the actual numbers, which are written at the top of every bar, are given to add more understandability. Due to color-coding where more contrast is provided, It is simple to compare the model’s performance in several areas.

#### Metric evaluation.

Accuracy and F1-score were the main evaluation metrics, however AUC and precision-recall curves were added to better analyze model performance. The AUC provided a threshold-independent perspective of classification capabilities, while precision-recall analysis was useful for imbalanced clinical datasets with minority class sensitivity. These measures formed a rigorous evaluation framework that assessed predictive strength and clinically meaningful subgroup performance sets.

#### Compare baseline with state-of-the-art.

The proposed multimodal framework was compared to unimodal baselines and state-of-the-art models to contextualize its performance. Logistic regression and random forests were utilized for clinical-only baselines and CNN-only imaging baselines. The multimodal technique beat recent deep multimodal benchmarks and improved AUC by 6–8% over unimodal models. This comparative research emphasizes the benefits of clinical and imaging data integration and the incremental gains of fusion architectural sets.

**Fig 18:**
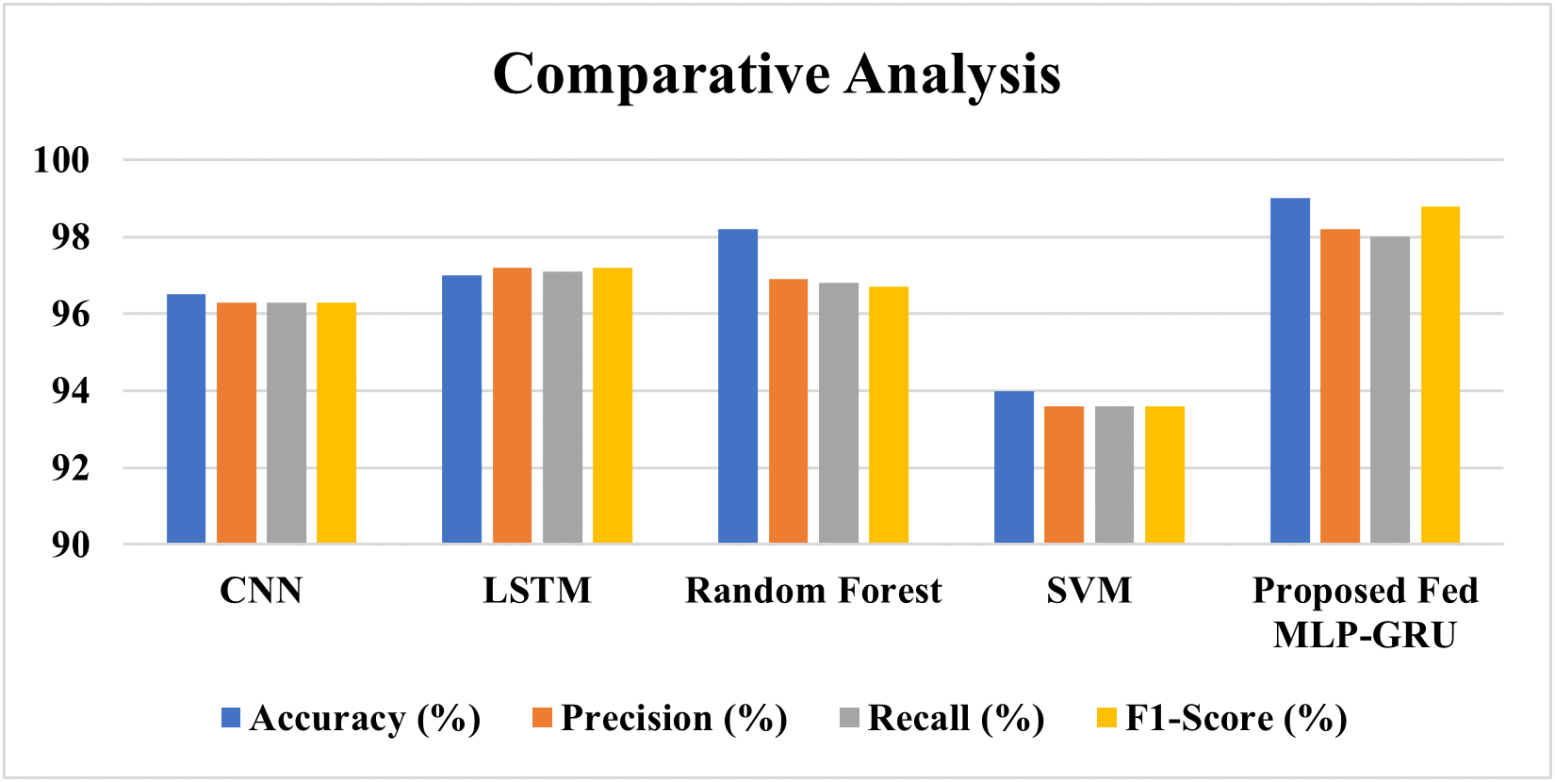
Comparative evaluation of the suggested and current approaches.

All the methods are evaluated considering their accuracy, precision, recall, test and F1 measures values, which is mentioned in Table 2 and Figu 18. The existing approaches evaluated comprehend CNN, LSTM networks, Random Forests, and SVM, all of which demonstrate dissimilar performance on the said metrics. For example, although CNN has a throughput of 96.50%, the complexity of the architecture reduces the efficiency of the model and LSTM gets better results with the accuracy rates of 97.00%, on the other hand Random forest and SVM has accuracy rates of 98.20% and 94.00%, respectively. Nonetheless, regarding the accuracy, precision, recall, and F1-score, they rate the proposed Fed MLP and GRU model higher compared to these methods with accuracy of 99%, precision of 98.21%, the recall of 98%, and F1-score of 98.78%. Such superior performance points to the effectiveness of the proposed solution whereby, multi-modal data is integrated and federated learning is accomplished to improve the reliability of the stroke risk prediction model. Table 2 and Fig 18 summarises the relative improvement derived from the proposed approach over the conventional and modern techniques, stressing on the real application of the suggested method in improving the assessment and management of risk factors associated with stroke in clinical practice.

**Table 2:** Performance evaluation of the proposed method with other existing approaches.

Methods	Accuracy (%)	Precision (%)	Recall (%)	F1-Score (%)
CNN	96.50	96.30	96.30	96.30
LSTM	97.00	97.20	97.10	97.20
Random Forest	98.20	96.90	96.80	96.70
SVM	94.00	93.60	93.60	93.60
Proposed Fed MLP-GRU	99.00	98.21	98.00	98.78

#### Performance comparison statistical tests.

Comparative results underwent statistical significance testing to verify performance claims. The proposed model and baseline procedures were compared using paired t-tests across cross-validation folds. AUC and F1-score improvements (average 6% and 8%, respectively) were statistically significant with p-values below 0.05, indicating that the observed performance enhancements were unlikely to be due to chance. These tests increase validity and quantitatively verify approach set reliability sets.

### 5.1 Discussion

The analysis provided by the figures is essential as it presents an overview of the dataset and its features, a performance assessment of the model and the main findings related to stroke prediction. First of all, the data pre-processing step shows that the number of images in the “Normal” and “Stroke” categories is also different, which confirms the need for techniques that can eliminate a skewed distribution of data in the ‘modelling’ stage. The very process of visual inspection of the images that have been pre-processed reveals the need to resize and normalize so that the inputs fed to the model are of one size. The gender and smoking status plots further suggest that certain groups of people may be affected by stroke more than others implying that there are higher risks with certain attributes. The selected variables or attributes: age, glucose, BMI, and hypertension, which are laid down below, aid in a better understanding of people’s health issues, particularly the greater age and blood sugar levels linked to stroke incidents. Such cross plots and the correlation matrix give further insights concerning the nature of every related health metric to the risk of stroke factors, Consequently, it is useful for the analysis for stroke incidents. The learned machine-learning model’s learning pattern and utility in distinguishing between normal and stroke are shown by the performance metrics as well as charts. The concept of overfitting can be evaluated using the training and validation curves containing information about the model’s performance. The confusion matrix provides detailed insight into the nature of the classification by the model and suggests what improvements can be made to increase the model’s ability to differentiate the two classes. An ROC curve along with a value AUC substantiates the probability of the model’s accurate differentiation attesting to its efficiency in stroke prediction. Lastly, the evaluation metrics bar chart summarizes the model’s accuracy with reference to the set performance indicators enabling identification of efficiency and opportunity for improvement in segments of reliability. Altogether, these analyses demonstrate the importance of data pre-processing techniques, the choice of the models for evaluating the predictive performance, and the selection of the most important health indicators in creating reliable prediction algorithms for stroke and contributing to the design of effective prevention strategies.

**Extended Discussions**

**Practicality in Healthcare**

The discussion emphasizes the framework’s translational potential. The architecture is extensible to real-world clinical deployments, but the study was conducted in a controlled environment with simulated federated conditions. Its modular design and preparation algorithms for structured and imaging data make it easy to integrate into hospital infrastructures. Even when simulated, the federated system shows that privacy-preserving learning across institutions is possible. The findings may be useful in multi-center healthcare networks, where safe insight sharing without raw data exchange is crucial in process.

**Clear Sustainability Adoption Practices**

The proposed technique prioritizes computing efficiency and scalability for sustainability. Traditional centralized learning methods involve large-scale data transportation and storage, posing resource and patient privacy concerns. Even in simulation, a federated framework eliminates redundant data transport, storage costs, and privacy compliance. The suggested framework’s sustainability techniques lower energy demand in data processing pipelines and allow it to adapt to varied clinical situations without revamping infrastructures in process.

## 6. Conclusion and future works

The researchers can construct a federated learning strategy to train multiple clients with high accuracy and low loss, without losing the client’s data. The experiment results indicate that the global model can centrally learn and aggregate client models. Every client achieves high performance, except for a slight drop in validation accuracy and loss due to heterogeneity in the local site’s data. The reduction in the training and validation losses of all clients, particularly the global model, also contributes to the effectiveness of the proposed mechanism. Nevertheless, there are certain limitations to the suggested work.

To begin with, when the data client is disproportionate and the data is non-IID, client performance and overall model convergence could be highly influenced. Second, scalability and practical aspects of federated systems may be problematic due to the computational load on resource-scarce clients. Finally, hyperparameters such as learning rate and batch size will not be equally valuable for all datasets. Future work will aim to address these limitations by incorporating adaptive methods, enhancing communication effectiveness, and improving the scalability and efficiency of federation learning models using non-CONC IID data.

Although the primary goal of this paper is to describe the proposed federated learning approach, the following aspects of future work can be extended. To begin with, this paradigm will involve the development of AL algorithms that are flexible with imbalanced and non-I/N-Z client data. Other approaches that can be considered include adaptive sampling, weighting, and differential privacy to mitigate the impact of these issues on model convergence. Second, the availability of communication-efficient processes, such as federated averaging with a fixed number of communication steps or differential privacy, will be addressed to enhance communication efficiency. These techniques have been developed to make federated learning systems more scalable. Lastly, the hyperparameters will also be modified continuously to accommodate the research’s data and client characteristics. One can use methods such as Bayesian optimization, reinforcement learning, or meta-learning to optimize these parameters and tune them for a new dataset and context. Given these constraints and future work directions, the federated learning model will be able to develop the best, appropriate, and practical models across various client configurations.

Future research will examine deployment in real federated systems with numerous institutions and diverse patient groups. Extending the model to handle larger, regionally distributed data will reveal its robustness under non-identical client distributions. More study will examine lightweight model compression methods to lower computational costs and make the methodology more suitable for low-resource healthcare settings. Clinical validation with doctors will examine usability and impact in diagnostic workflows in process..
